# Metal–Organic Framework‐Preserved Thermostable Microneedle Patch for Minimally Invasive Detection and Monitoring of Kidney Dysfunction

**DOI:** 10.1002/adma.74423

**Published:** 2026-08-05

**Authors:** Yixuan Wang, Ying Liu, Avishek Debnath, Chuang Li, Zheyu Wang, Gayathri Kondepati, Saketh Balmoori, Jeremiah J. Morrissey, Ying Maggie Chen, Limei Tian, Srikanth Singamaneni

**Affiliations:** ^1^ Department of Mechanical Engineering and Materials Science Institute of Materials Science and Engineering Washington University in St. Louis St. Louis Missouri USA; ^2^ Department of Biomedical Engineering Center For Remote Health Technologies and Systems College Station Texas A&M University Texas USA; ^3^ Division of Nephrology Department of Internal Medicine Washington University School of Medicine St. Louis Missouri USA; ^4^ St. Louis Veteran Affairs Medical Center St. Louis Missouri USA

**Keywords:** kidney, LPS‐induced AKI, metal–organic framework (MOF), microneedle, minimally‐invasive in vivo detection, preservation

## Abstract

Early and decentralized biomarker detection and monitoring is essential for timely diagnosis and intervention, particularly in kidney disorders that often progress asymptomatically until irreversible damage has occurred. Here, we present a thermally resilient metal‐organic framework encapsulated microneedle (MOF@MN) sensing platform that integrates minimally invasive dermal interstitial fluid (ISF) sampling and on‐needle detection with MOF‐based biostabilization of the sensing interface. A thin zeolitic imidazolate framework‐8 (ZIF‐8) shell is grown in situ on antibody‐functionalized microneedles, preserving the bioactivity of immobilized antibodies after 4 weeks of thermal cycling up to 50°C and maintaining full analytical performance after prolonged unrefrigerated shipment. This interfacial encapsulation strategy enables quantitative detection of neutrophil gelatinase‐associated lipocalin (NGAL), an early biomarker of kidney injury, directly from dermal ISF over a broad clinically relevant range. In a mouse model and human subjects, MOF@MN‐derived NGAL measurements closely mirror blood NGAL concentrations, precede changes in conventional renal function markers, and correlate well with histopathological injury severity, highlighting their potential for subclinical monitoring. Collectively, this study establishes conformal MOF encapsulation as a simple and highly effective strategy for engineering environmentally resilient sensing interfaces and provides a scalable route to cold‐chain‐independent, minimally invasive biosensing for decentralized and at‐home health monitoring.

## Graphical abstract

By growing a conformal ZIF‐8 protective layer directly on antibody‐functionalized microneedles, this work converts a fragile biosensing interface into a thermostable diagnostic platform. The MOF@MN patch enables minimally invasive NGAL detection from dermal ISF while supporting cold‐chain‐independent storage and transport for decentralized kidney‐dysfunction monitoring.

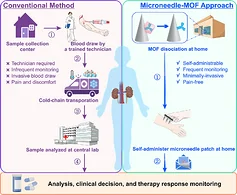

## 1 Introduction

Timely and accurate detection of disease‐associated biomarkers is essential for early diagnosis, therapeutic monitoring, and prevention of disease progression in a wide range of clinical conditions [1]. Kidney diseases, both acute and chronic, pose a significant global health challenge, often progressing asymptomatically until reaching an advanced stage where treatment options are limited and outcomes are poor [2, 3]. These conditions contribute to millions of deaths annually and place significant strain on healthcare systems worldwide [4]. Early detection and frequent monitoring are therefore critical for improving clinical outcomes [5]. However, current diagnosis of kidney dysfunction relies heavily on conventional indicators such as serum creatinine (sCr) and blood urea nitrogen (BUN), which suffer from delayed responsiveness and low specificity [6, 7]. These conventional markers are influenced by a range of confounding physiological variables, including hydration status, age, muscle mass and comorbidities, thereby limiting their utility for detecting early onset of disease [6, 7, 8].

To improve early and accurate diagnosis, attention has shifted toward protein biomarkers that respond more sensitively and rapidly to kidney stress [9]. For example, neutrophil gelatinase‐associated lipocalin (NGAL), a 25 kDa glycoprotein upregulated within hours of tubular injury, has emerged as a clinically validated marker for renal damage [6, 8, 10]. However, NGAL quantification in clinical settings remains dependent on centralized laboratory infrastructure, invasive sample collection, and cold‐chain logistics, limiting its utility in resource‐limited settings, point‐of‐care testing and home‐based monitoring (Figure 1) [1, 11]. These limitations are especially burdensome for pediatric, geriatric, and chronically ill populations, for whom frequent phlebotomy increases infection risk, discomfort, and reduced compliance. There is a pressing need for diagnostic platforms that enable decentralized, minimally invasive monitoring of clinically relevant biomarkers in a patient‐friendly manner.

**FIGURE 1:**
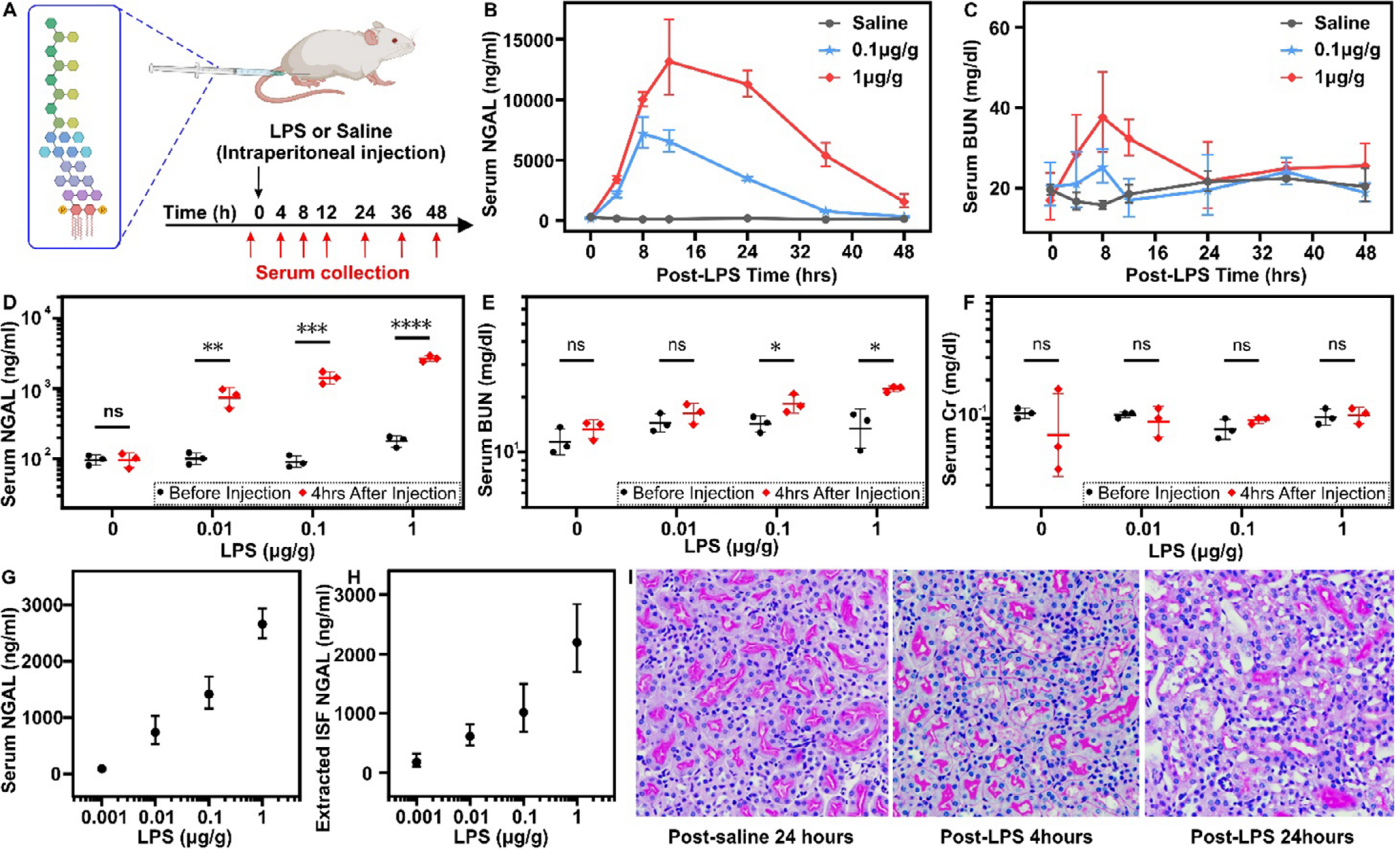
Dose‐dependent and temporal profiles of renal injury biomarkers in response to LPS‐induced acute kidney injury (AKI). (A) Schematic illustration of the experimental timeline for intraperitoneal injection of lipopolysaccharide (LPS) at varying doses (0, 0.01, 0.1, and 1 µg/g body weight) in BALB/C mice. Serum was collected at different points pre‐ and post‐injection. (B,C) Temporal dynamics of serum neutrophil gelatinase‐associated lipocalin (NGAL) and blood urea nitrogen (BUN) at 0.1 and 1 µg/g LPS dosages compared with saline controls. (D–F) Dose‐dependent comparison of serum NGAL, BUN, and serum creatinine (sCr) levels before and 4 h after LPS administration, demonstrating significant NGAL elevation even at lower doses of LPS. Statistical significance was determined using unpaired t‐tests (*ns* = non‐significant; *p < 0.05, **p < 0.01, ***p<0.001, ****p < 0.0001). (G,H) Serum and extracted interstitial fluid (ISF) NGAL concentrations showing a robust, dose‐dependent increase, suggesting ISF as a feasible, less invasive alternative biofluid for NGAL measurement. (I) Periodic acid‐Schiff staining of renal tissue collected 4 and 24 h after saline or LPS administration. Error bars represent mean ± standard deviation (SD). *n* = 3 mice per group for each condition.

Microneedle (MN)‐based biosensors offer a promising alternative by enabling minimally invasive, real‐time access to interstitial fluid (ISF) [12, 13, 14, 15], a clinically informative biofluid that mirrors systemic biomarker levels [16, 17]. Microneedles penetrate only the outermost skin layer (stratum corneum), avoiding pain, bleeding, and the need for trained personnel. MNs can be functionalized to capture target analytes in situ, simplifying the workflow by eliminating the need for biofluid extraction [12]. Various microneedle designs have been explored for potential clinical applications, including solid microneedles, coated microneedles, hydrogel‐based microneedles, hollow microneedles, and dissolvable microneedles [18, 19]. Our prior work has established polystyrene‐based microneedle patches combined with plasmonic fluor as nanolabels for ultrasensitive detection of low‐abundance protein biomarkers [20]. Adapting this MN technology for renal biomarker monitoring poses two critical challenges. First, renal injury biomarkers such as NGAL can span several orders of magnitude during disease progression, requiring biosensors with both high sensitivity and broad dynamic range [9, 10]. Second, the surface‐bound biorecognition elements on MNs are vulnerable to thermal and environmental stress, creating storage and transport constraints that limit use in at‐home, decentralized, or resource‐limited settings where cold‐chain logistics may be unreliable or unavailable and ambient temperature can reach above 50°C [21, 22].

Metal‐organic frameworks (MOFs), a class of crystalline, porous materials formed through the coordination of metal ions with organic linkers, have shown considerable potential for stabilizing biomolecules under environmental stress [23, 24, 25, 26, 27]. Among them, zeolitic imidazolate frameworks (ZIFs) offer several attractive properties, including tunable pore size, aqueous synthesis, excellent biocompatibility, and exceptional thermal and chemical stability [28, 29, 30]. Although MOFs have been explored for stabilizing soluble biomolecules, conformal encapsulation of biomolecules on complex surfaces, such as biofunctionalized microneedles, remains unexplored.

Herein, we present a novel MOF‐encapsulated microneedle (MOF@MN) biosensing platform for robust, quantitative, minimally invasive, and field‐deployable detection of kidney disease biomarkers from dermal ISF (Scheme 1). This MN‐based platform addresses a central limitation of existing microneedle‐based diagnostics by overcoming the intrinsic instability of biorecognition molecules under environmental stress. We harness a conformal in situ ZIF‐8 encapsulation that preserves bioactivities of MN‐surface‐bound antibodies under prolonged thermal and environmental exposure while maintaining molecular accessibility for target recognition. This encapsulation strategy enables cold‐chain‐independent operation and extends biosensor applicability to decentralized and resource‐limited environments.

**SCHEME 1:**
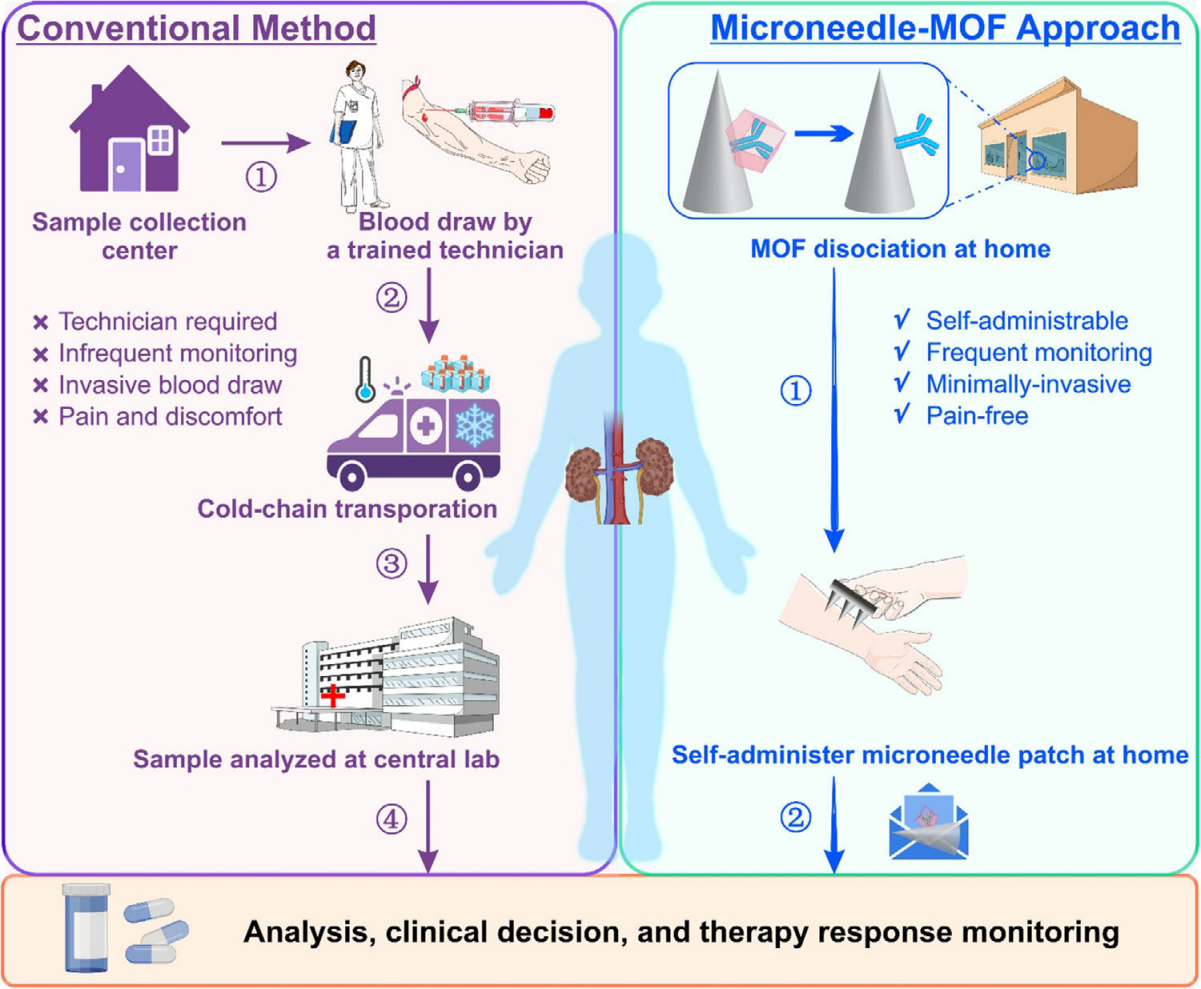
Schematic illustration depicting MOF‐encapsulated microneedle biosensing versus conventional blood draw‐based diagnosis and monitoring of acute kidney injury.

## 2 Results

### 2.1 Early Inflammatory and Renal Response to LPS Administration: NGAL as a Quantitative Marker of Kidney Injury

Lipopolysaccharide (LPS) administration is widely used to simulate key features of sepsis‐associated acute kidney injury (AKI) in a mouse model, including inflammation and oxidative stress mediated through Toll‐like receptor 4 (TLR4) signaling [31, 32, 33]. Following TLR4 activation, renal tubular epithelial cells rapidly upregulate and secrete NGAL as part of the acute injury response [31, 32, 34]. By profiling NGAL kinetics alongside conventional kidney function markers such as blood urea nitrogen (BUN) and serum creatinine (sCr), we aimed to elucidate the extent and temporal progression of renal injury in this model.

We evaluated the time‐dependent and dose‐dependent responses of serum NGAL levels in BALB/C mice following intraperitoneal injection of LPS at doses of 0.1 µg/g (moderate dose) and 1 µg/g body weight (high dose), with saline‐injected mice serving as the control group (Figure 1A). Time‐course analysis revealed rapid and dose‐dependent elevations in serum NGAL concentrations, peaking at approximately 8–12 h post‐LPS injection. At moderate and high LPS dose, NGAL concentrations exceeded 8000 ng/mL, significantly higher than the saline group (100–300 ng/mL) (Figure 1B). In contrast, BUN and sCr exhibited a delayed and modest increase, even at the highest dose (Figure 1C; Figure S1). To further investigate the applicability of various biomarkers in the early detection of AKI, we compared serum NGAL, serum BUN, and serum sCr concentrations at 4 h post‐LPS injection across the three dosing groups (low dose at 0.01 µg/g, moderate dose at 0.1 µg/g, and high dose at 1 µg/g body weight) (Figure 1D–F). NGAL concentrations were significantly elevated even at the lowest LPS doses (0.01 µg/g), whereas BUN displayed no significant early changes at the low dose and sCr showed no significant changes at all doses, highlighting the superior sensitivity of NGAL in detecting subclinical kidney injury in this model.

Furthermore, dose‐response analyses confirmed a strong correlation between administered LPS dose and NGAL levels measured in both serum and extracted interstitial fluid (ISF), indicating that NGAL concentration reliably reflects the severity of inflammation‐induced renal damage (Figure 1G,H). Histopathological examinations provided complementary evidence (Figure 1I): Periodic acid‐Schiff (PAS) staining revealed early and localized tubular damage as early as 4 h post‐LPS injection, characterized by brush border detachment from proximal epithelial cells. By 24 h post‐LPS injection, these morphological changes became more pronounced, including significant loss of brush borders, partial cytoplasmic degeneration, and prominent tubular dilation, clearly indicative of progressing kidney injury (Figure 1I) [35, 36, 37]. These structural alterations correlated well with the observed NGAL elevations, confirming the sensitivity in reflecting renal tissue damage at the early stage. Collectively, these results confirm that NGAL serves as a sensitive and effective marker for tracking renal injury progression in the LPS‐induced AKI model, with a rapid and dose‐dependent response that precedes changes in conventional kidney markers, such as BUN and sCr. The observed dynamics of various biomarkers, including NGAL, lay the critical groundwork for developing minimally invasive biosensing platforms that aim to improve early diagnosis, timely intervention, and personalized management of AKI.

### 2.2 Microneedle‐Based Quantification of NGAL in a Mouse Model

To enable minimally invasive and quantitative detection of NGAL from dermal interstitial fluid (ISF), we established a fluorophore‐linked immunosorbent assay (FLISA) directly on polystyrene microneedle (MN) patches, designed for in situ biomarker capture and ex vivo quantification (Figure 2A). Conventional enzyme‐linked immunosorbent assays (ELISA) [38] are unsuitable for microneedle platforms due to their limited reaction volume and low surface area, restricting colorimetric signal development and readout sensitivity [20]. Alternatively, fluorescence‐based strategies enable the acquisition of spatially resolved, on‐needle signals with high sensitivity and stability. The key analytical challenge for NGAL is achieving an extended dynamic range and quantification precision. Here, we employed IRDye 800CW‐labeled streptavidin to achieve a robust fluorescence signal and accurate quantification across a wide physiological concentration range of NGAL (Figure 2A–C).

**FIGURE 2:**
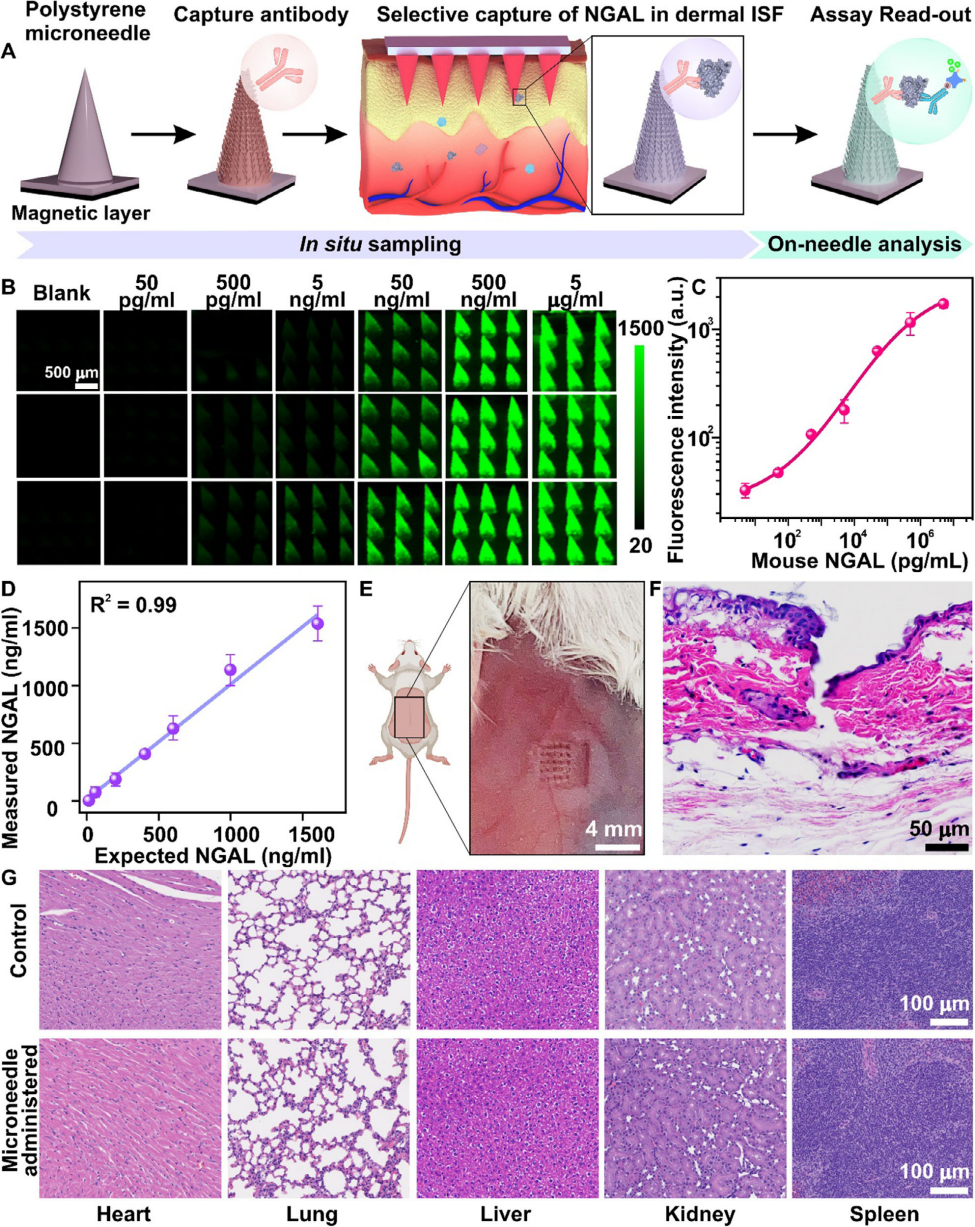
MN‐enabled NGAL quantification in mouse ISF. (A) Schematic of the MN‐based NGAL detection workflow, illustrating in situ NGAL capture in dermal interstitial fluid (ISF) and on‐needle analysis. (B) Fluorescence intensity maps of MN patches following exposure to varying concentrations of mouse NGAL, confirming a dose‐dependent signal response. (C) Standard curve illustrating the extended dynamic range of NGAL detection, showing a dose‐dependent fluorescence intensity on MN. (D) Linearity analysis highlighting strong correlation (*R* ^2^ > 0.99) between expected NGAL concentration and NGAL concentration measured through MN patches. (E) Optical image of mouse ventral skin after MN patch administration. (F) H&E‐stained cross‐section of mouse skin confirming the penetration by a single microneedle. (G) H&E‐stained sections of mouse major organs with and without administration of MN patches, indicating excellent biocompatibility of MN patches.

Polystyrene MN patches were fabricated using a two‐step drop‐casting method, resulting in a bilayer structure with a magnetic nanoparticle‐infused backing for high‐throughput and reproducible immunoassay processing in standard 24‐well plates. The resulting MN patches comprise an 11 × 11 array of conical MN (629 ± 10 µm height, 300 µm base diameter, 5 µm tip radius, and tip‐to‐tip spacing of 600 µm) (Figure 2B; Figure S2). Prior to designing MN‐based assay, we first validated the uniformity of antibody immobilization on the MN by immobilizing biotinylated antibodies on the microneedle surface, followed by blocking with bovine serum albumin (BSA) and subsequently incubating with IRDye LT680‐labelled streptavidin. Confocal fluorescence images indicated a uniform distribution of the antibody along the microneedle length, confirming effective and homogeneous antibody coating (Figure S3). To construct the MN‐based FLISA, NGAL capture antibodies were immobilized on the polystyrene surface via hydrophobic interactions and blocked with BSA to minimize nonspecific interactions [39]. The resulting functionalized MN patches were exposed to recombinant NGAL protein (ex vivo) or administered on mouse skin (in vivo), where MN selectively captures NGAL in the local dermal ISF in a concentration‐dependent manner. After 1 min of administration, MN patches were peeled off from the skin, and the NGAL bound on MN was quantified by an ex vivo immunoassay involving the incubation with biotinylated detection antibodies and IRDye 800CW‐streptavidin (Figure 2A). The formation of the fluorescently labeled immunocomplex on the MN generates a homogeneous fluorescence signal along the MN (Figure 2B). Control patches coated with BSA alone exhibited a negligible background signal (Figure S4), indicating a negligible auto‐fluorescence from BSA and polystyrene MN and minimal nonspecific binding of the dye‐labeled streptavidin.

To evaluate the analytical performance of the MN‐based NGAL assay, we established a standard calibration curve using serial dilutions of recombinant NGAL protein ranging from blank (0 pg ml^−1^) to 5 µg ml^−1^ in 1% BSA‐PBS buffer. The assay achieved a limit of detection (LOD) of 16.3 pg ml^−1^, calculated as the blank signal plus three times the standard deviation (mean + 3σ of blank) (Figure 2C). To validate the quantification accuracy of the assay, we compared the measured NGAL concentration from MN patches exposed to known concentrations of NGAL spiked in diluted mouse ISF matrix against the expected concentrations from the standard curve. The measured values closely matched the expected concentrations across the dynamic range, demonstrated excellent linearity across a broad dynamic range (0.1–2000 ng ml^−1^) with *R* ^2^ > 0.99 (Figure 2D; Figure S5), confirming the robust analytical precision of the on‐needle detection and quantification.

Histological examination (Hematoxylin and eosin (H&E)‐staining) validated MN penetration depths of 125–165 µm into dermis (Figure 2E,F), effective for accessing ISF while avoiding vascular injury (Figure S6). To further assess potential blood‐derived contamination, we examined MN surface morphology after MN skin administration. The standard MN (629 ± 10 µm height) used for NGAL detection retained intact MN morphology after administration and showed no evident blood‐cell‐like structures on the MN surface. In contrast, the 1.5 mm height MN patch, used as a positive control for deeper penetration into dermal tissue, showed numerous round cell‐like features on the MN surface after skin administration (Figure S7). These results support that the standard MN geometry and administration procedure used for NGAL detection are unlikely to introduce blood‐derived contamination. To understand whether shear forces from skin insertion affect antibody stability, we administered anti‐NGAL coated MN patches to the ventral skin for 5 min. Following the removal of MN patches from skin, we performed FLISA on MN by exposing pristine and skin‐pierced MN patches to recombinant mouse NGAL proteins with 500 ng ml^−1^. The fluorescence intensity of skin‐pierced MN was comparable to that of pristine MN patch, indicating that the antibody‐coated MN retained antigen‐binding capacity after repeated insertion (Figure S8). Biocompatibility assessments did not reveal observable histological alterations in vital organs (heart, lungs, liver, kidneys, and spleen) following MN administration, indicating minimal systemic toxicity under tested conditions (Figure 2G). These results are consistent with our previous characterization of the same polystyrene antibody‐functionalized MN system, in which human dermal fibroblast (HDF) viability remained unchanged after 1 or 16 h of exposure to pristine and BSA‐coated MN patches [20]. Furthermore, saline‐injected control mice that received MN administration for 20 mins showed no statistically significant elevation in dermal ISF IL‐6. Collectively, these results validate the polystyrene MN patches as an effective, structurally stable, biocompatible and analytically reliable platform for fluorescence‐based NGAL detection and quantification from dermal ISF.

### 2.3 Detection and Quantification of NGAL in Dermal ISF of Human Subjects

To extend the MN biosensing platform from preclinical validation to human application, we evaluated its analytical performance and biocompatibility in healthy volunteers by quantifying NGAL concentrations in dermal ISF and comparing them with matched plasma concentrations. This experiment aimed to confirm the reliability, reproducibility, and translational feasibility of MN‐based NGAL quantification under minimally invasive conditions. Prior to human testing, NGAL antibody‐functionalized MN patches were assessed for endotoxin content and contained 0.085 EU per device (Figure S9), well below the U.S. Food and Drug Administration (FDA) safety threshold of 20 EU per device for materials contacting vascular or lymphatic systems, supporting the device's safety profile with respect to endotoxin levels.

To obtain consistent skin penetration and signal acquisition, a spring‐loaded applicator was employed for MN patch deployment (Figure 3A–C). The applicator's central loading platform ensured uniform force distribution and precise insertion depth across the MN array. The antibody‐functionalized MN patch was first mounted onto the applicator loading center (Figure 3C1,C2). Then, the MN‐loaded applicator was applied to the forearm to enable NGAL capture from dermal ISF (Figure 3C3,C4). After 5 min, the MN patch was removed, revealing uniform micropores without bleeding, and the skin fully recovered within 30 min (Figure 3D). Optical microscope image confirmed that MNs remained structurally intact after administration (Figure 3E), demonstrating their mechanical robustness. The standard curve of MN‐based NGAL assay was performed by exposing antibody‐functionalized MN patches to serial dilutions of recombinant NGAL protein for 5 min, followed by detection antibody and labeling steps. The assay achieved an LOD of 9 pg ml^−1^, demonstrating high analytical sensitivity across physiologically relevant ranges (Figure 3F).

**FIGURE 3:**
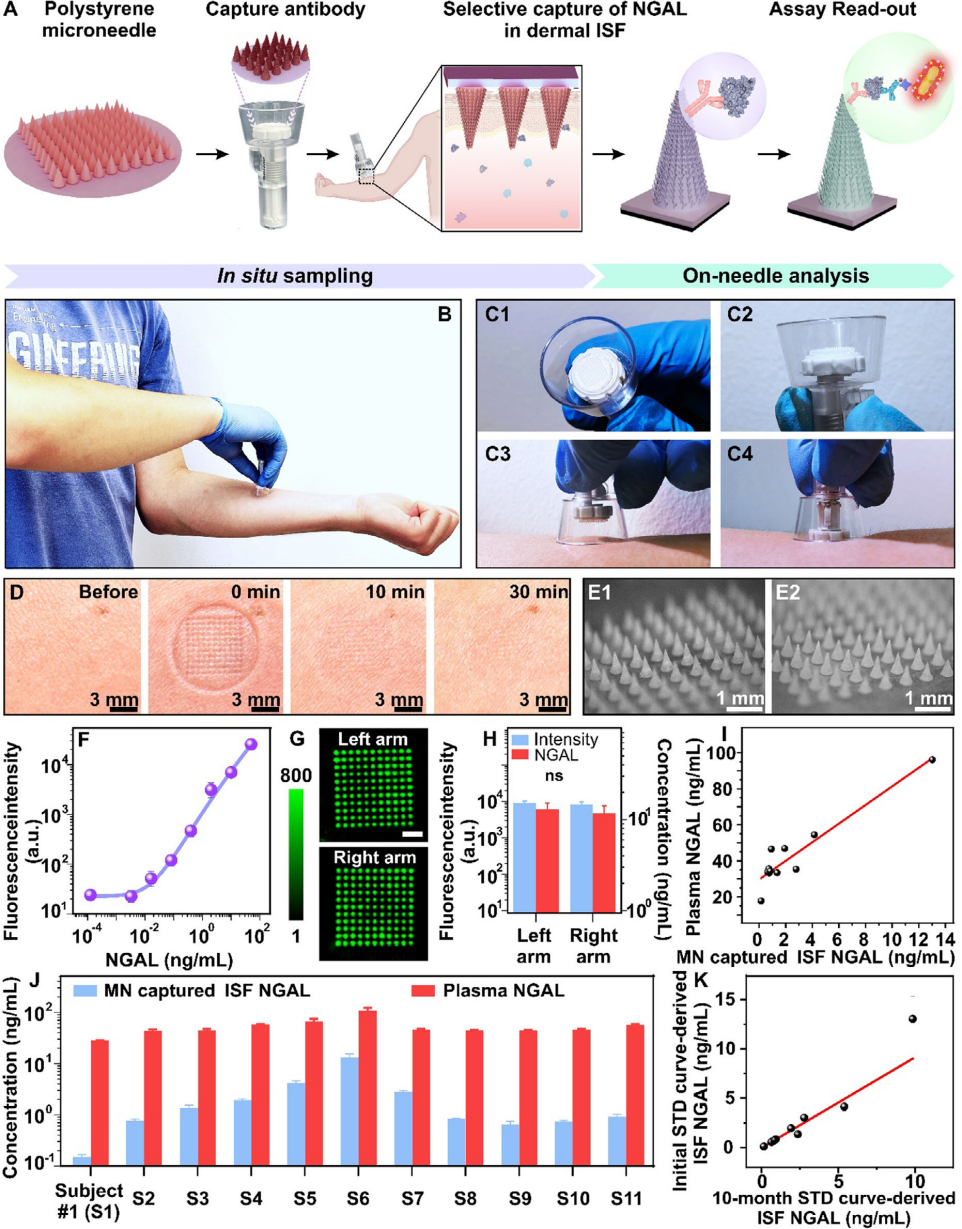
MN‐enabled NGAL quantification in human ISF. (A) Schematic of the human NGAL detection workflow, illustrating in situ NGAL capture in dermal ISF and on‐needle analysis. (B) Optical image of a human subject self‐applying an MN patch using a spring‐triggered applicator. (C) Top view (C1) and side view (C2) optical images of an MN patch loaded on the applicator. Optical images of the applicator positioned on the skin before (C3) and after (C4) releasing the MN patch. (D) Skin recovery following microneedle administration. (E) Morphology of microneedle tips before (E1) and after (E2) administration on human skin. (F) Standard curve of human NGAL plasmonic fluor‐linked immunosorbent assay (p‐FLISA) on the microneedle. (G) Fluorescence images of MN patches following administration on human subject forearms (Scale bar: 1500 µm), and (H) corresponding fluorescence intensity and NGAL concentration. Statistical significance was determined using unpaired *t*‐tests (ns = nonsignificant). (I) Correlation between NGAL levels in human ISF and plasma (*n* = 11). (J) Comparison of NGAL concentrations measured in ISF and plasma from 11 human subjects. (K) Correlation of NGAL concentrations quantified using independent calibration curves generated 10 months apart. The concentration derived from the two calibration curves showed strong agreement.

To evaluate measuring consistency, MN patches were applied to the left and right forearms of a human subject. Fluorescence imaging displayed uniform signal distribution across the 11 × 11 MN patches from different arms, with no statistically significant difference in quantified NGAL concentrations (Figure 3G,H), confirming the consistency of MN‐based quantification across skin sites. To assess intersubject reproducibility and biological correlation with plasma NGAL level, MN patches were applied to the forearms of three healthy subjects, and plasma samples were collected in parallel by finger prick. Plasma NGAL concentrations were higher than MN‐captured dermal ISF NGAL concentrations, with mean (*n* = 11) levels of 52.5 ± 20.2 ng mL^−^^1^ in plasma and 2.40 ± 2.88 ng mL^−^^1^ in dermal ISF. Despite this difference in absolute concentration, the two measurements showed a strong positive correlation, with a Pearson correlation coefficient of *R* = 0.94 (Figure 3I,J). This observation indicates that MN‐captured ISF NGAL can effectively reflect the variations in systemic NGAL levels, although the ISF and plasma concentrations are not quantitatively identical. To further evaluate analytical reproducibility across experimental batches, human MN samples were cross‐analyzed using independent MN‐FLISA calibration curves generated 10 months apart. NGAL concentrations calculated from the two temporally separated calibration batches showed excellent agreement, with a Pearson correlation coefficient of *R* = 0.9725 (Figure 3K), supporting robust inter‐batch calibration consistency and reliable concentration assignment for MN‐based NGAL quantification.

### 2.4 In Situ MOF Encapsulation for Enhanced Stability of Antibody‐Functionalized Microneedle Patches

Following validation of MN‐based NGAL quantification in both murine models and human subjects, the next key translational hurdle is the stability of antibody‐functionalized MN patches. Antibody coatings are inherently prone to thermal and temporal degradation, restricting their utilization beyond controlled refrigeration environments. However, practical deployment in home‐based monitoring, decentralized care, and resource‐limited settings demands devices that can retain functionality over extended periods without relying on cold‐chain infrastructure. To address this limitation, we investigated in situ growth of a protective ZIF‐8 layer on the antibody‐functionalized MN patches (Figure 4A), aiming to preserve bioactivity without compromising detection performance.

**FIGURE 4:**
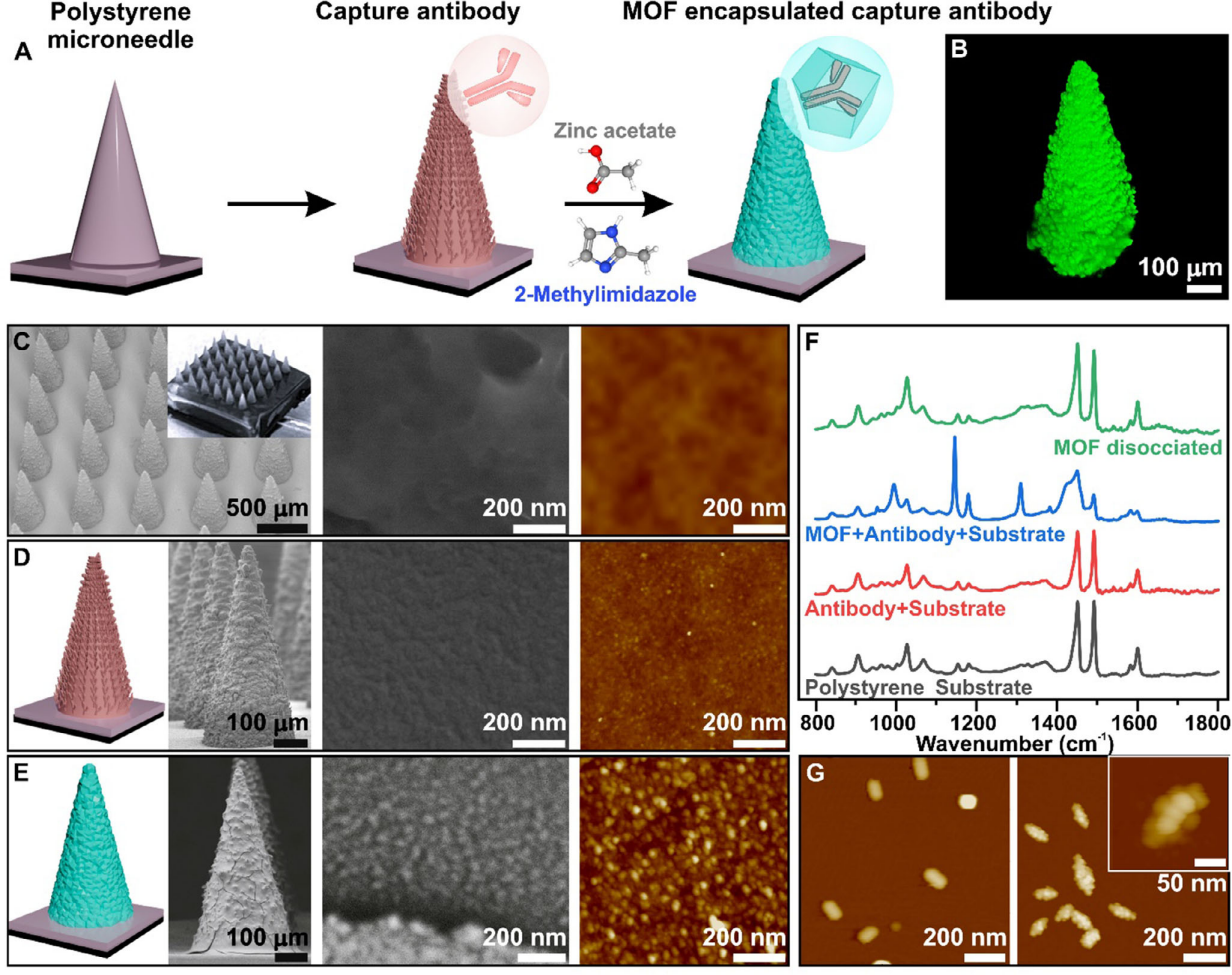
In situ growth of ZIF‐8 on NGAL capture antibody‐functionalized microneedle. (A) Schematic illustration of the in situ growth of ZIF‐8 on MN, where a polystyrene microneedle is first functionalized with capture antibodies, followed by in situ growth of ZIF‐8. (B) Confocal fluorescence microscopy image confirming uniform adsorption of antibodies on polystyrene MN. (C–E) SEM and AFM images of microneedle surfaces before and after ZIF‐8 encapsulation, (C) pristine polystyrene surface, (D) capture antibody functionalized polystyrene surface, (E) ZIF‐8‐encapsulated functionalized microneedle, demonstrating progressive morphological changes. (F) ATR‐FTIR spectra confirming the formation of ZIF‐8 and its interaction with antibodies‐functionalized surface. (G) High‐resolution AFM images showing 30 ± 5 nm ZIF‐8 nanocrystals continuously in situ growth on antibody‐functionalized gold nanorods, demonstrating uniform coverage. Inset scale bar, 50 nm. SEM image of (C–E) performed on microneedle surface, AFM images of (C–E) performed on a flat polystyrene‐coated silicon wafer, replicating the surface chemistry of MN.

ZIF‐8 encapsulation of the antibody‐functionalized MN surface was achieved by exposing the MN patch to an aqueous solution comprising zinc acetate and 2‐methylimidazole. This strategy leveraged abundant nucleation sites provided by the immobilized antibody, facilitating localized, conformal crystal growth (Figure 4A) (see Method Section for details). The encapsulation process resulted in a continuous ZIF‐8 protective layer that directly formed around immobilized antibodies, tightly preserving their structural integrity and biorecognition ability. Scanning electron microscopy (SEM) and atomic force microscopy (AFM) revealed the morphological changes of the MNs after ZIF‐8 encapsulation (Figure 4C,E). Although pristine polystyrene MNs displayed smooth surfaces, antibody‐functionalized MNs presented increased nanoscale surface roughness. After ZIF‐8 in situ growth, MN surfaces transitioned to uniform granular textures indicative of continuous ZIF‐8 crystal coverage. An identical procedure was replicated on a flat polystyrene film coated on a silicon substrate to further understand the morphological changes. Corresponding AFM imaging confirmed uniform ZIF‐8 nanocrystal coverage (Figure 4E), with ZIF‐8 protective layer thickness of 27 ± 4 nm (Figure S10).

Attenuated total reflection Fourier‐transform infrared (ATR‐FTIR) spectroscopy further confirmed the successful growth of ZIF‐8 (Figure 4F). The pristine polystyrene and antibody‐functionalized surfaces displayed characteristic polymer backbone signatures, including aromatic C─H bending at 905 cm^−^^1^, CH_2_ scissoring vibration at 1451 cm^−^^1^, and aromatic C═C stretching at 1492 and 1600 cm^−^^1^ (Figure 4F) [40]. After ZIF‐8 encapsulation, new absorption bands appeared at 1146 and 1179 cm^−^^1^, corresponding to C─N stretching of the imidazolate linker coordinated to Zn^2+^, with imidazole ring vibrations at 995 and 1310 cm^−^^1^ [41, 42]. The convoluted peaks in 1382–1491 cm^−^^1^ region were attributed to ring stretching modes, collectively confirming the in situ formation of the ZIF‐8 [29, 42, 43]. Upon dissociation of ZIF‐8 protective layer using an EDTA/phosphate buffer solution (pH ≈ 5.4), the FTIR spectra reverted to their original antibody‐functionalized polystyrene profiles, confirming the complete and reversible encapsulation without chemical alteration of the functionalized substrate (Figure 4F). Complementing FTIR analysis, we acquired Raman spectra to further validate the structural evolution during ZIF‐8 growth and removal. The pristine polystyrene surface exhibited characteristic bands at 795 cm^−^^1^ (aromatic C─H out‐of‐plane deformation) and 1001 cm^−^^1^ (aromatic ring breathing mode) (Figure S11) [44]. Following ZIF‐8 encapsulation, new bands appeared between 1202 and 1420 cm^−^^1^, corresponding to imidazole ring vibrations and C─N stretching modes [45], confirming the formation of ZIF‐8 crystals. Upon removal of the ZIF‐8 layer, Raman spectra resembled the original polystyrene surface profile, indicating complete ZIF‐8 dissociation without detectable residues.

We employed IgG‐conjugated gold nanorods (IgG‐AuNRs) as nanoscale templates to reveal the finer details of the ZIF‐8 nanocrystal encapsulation of the biomolecules. AFM analysis revealed uniform and grainy ZIF‐8 nanocrystals (30 ± 5 nm in diameter) surrounding IgG‐AuNR conjugates (Figure 4G). This morphology differs substantially from the characteristic rhombic dodecahedral ZIF‐8 crystals, 300–500 nm in size, that form on MN surfaces under different synthesis conditions [46]. The difference in the crystal dimension and morphology suggests two distinct nucleation pathways. Under the optimized encapsulation conditions used in this study, moderate supersaturation and a balanced 2‐methylimidazole‐to‐zinc ratio favor biomolecule‐mediated heterogeneous nucleation. The abundant functional groups on the antibodies provide numerous heterogeneous nucleation sites, generating small ZIF‐8 nuclei that grow into nanoscale crystals, which coalesce into a thin, continuous, and conformal nanocrystalline shell [47, 48]. In comparison, the more concentrated, ligand‐rich precursor promotes homogeneous nucleation in the bulk solution, followed by rapid crystal growth and ripening that are weakly guided by the biomolecular surface [47, 49]. These structural differences are consistent with the preservation results (Figures S12–S14). We attribute the improved preservation under the optimized condition to both the conformality and the protein‐templated nature of the ZIF‐8 coating. A nanometer‐thick MOF shell formed directly around the immobilized antibody interface can physically isolate the bioactive layer from the external environment, restrict large‐scale antibody conformational changes, suppress aggregation or interfacial unfolding, and reduce dehydration‐induced structural perturbation during refrigeration‐free storage and transportation. Such confinement‐based stabilization is consistent with the high preservation efficacy observed under the optimized MOF encapsulation condition. In contrast, although larger bulk‐grown crystals can also form an apparent continuous layer, their formation is less biomolecule‐templated and requires greater interfacial reorganization, which correlates with reduced antibody stability under thermal stress (Figures S12–S14).

We measured the endotoxin level of the MN patches after ZIF‐8 encapsulation and removal process, matching the final state used for in vivo application. The measured endotoxin level was 0.05 EU per MN patch, well below the FDA safety threshold (20 EU per device) (Figure S15, Table S1). The results confirm that the MOF in situ growth and dissociation procedure introduces negligible endotoxin contamination, supporting the suitability of MOF‐preserved MN patches for in vivo applications. Collectively, these morphological and spectroscopic analyses underscore the effectiveness of ZIF‐8 encapsulation strategy in achieving conformal, structurally robust protective coating for preserving antibody‐functionalized MN surfaces. This preservation technique significantly enhances antibody stability under harsh conditions, supporting reliable, minimally invasive biomarker detection in diverse, real‐world diagnostic scenarios.

### 2.5 Thermal and Temporal Stability of MOF‐Encapsulated Microneedle Patches Under Environmental Stress

To evaluate the preservation performance of the MOF‐encapsulated microneedle (MOF@MN) patches under thermal stress, we first assessed whether the ZIF‐8 growth and removal process itself would compromise assay sensitivity. NGAL capture antibody‐functionalized MN were encapsulated with ZIF‐8 and immediately subjected to rapid dissociation using an EDTA‐phosphate buffer (Figure 5A). Following dissociation, NGAL FLISA was performed directly on the MN patches to assess analytical performance after treatment (Figure 5B). We generated standard curves from three groups of MN patches: freshly prepared MN patches, MN patches coated with sucrose and stored at −20°C for 1 week, and MN patches subjected to ZIF‐encapsulation and immediate dissociation (i.e., no storage time). Fluorescence intensities were normalized against the blank intensity of each condition, enabling direct and accurate comparison of assay performance across experimental groups (Figure 5B). The resulting standard curves exhibited excellent consistency (in terms of slope, dynamic range of the fluorescence signal, and analyte concentration) compared to those from a freshly performed microneedle assay, indicating that neither MOF encapsulation nor the dissociation process compromised antibody bioactivity or detection sensitivity. MN patches coated with sucrose and stored at −20°C for 1 week generated the standard curve that was identical to freshly prepared MN patches, therefore this storage condition was selected as the positive control in all subsequent experiments.

**FIGURE 5:**
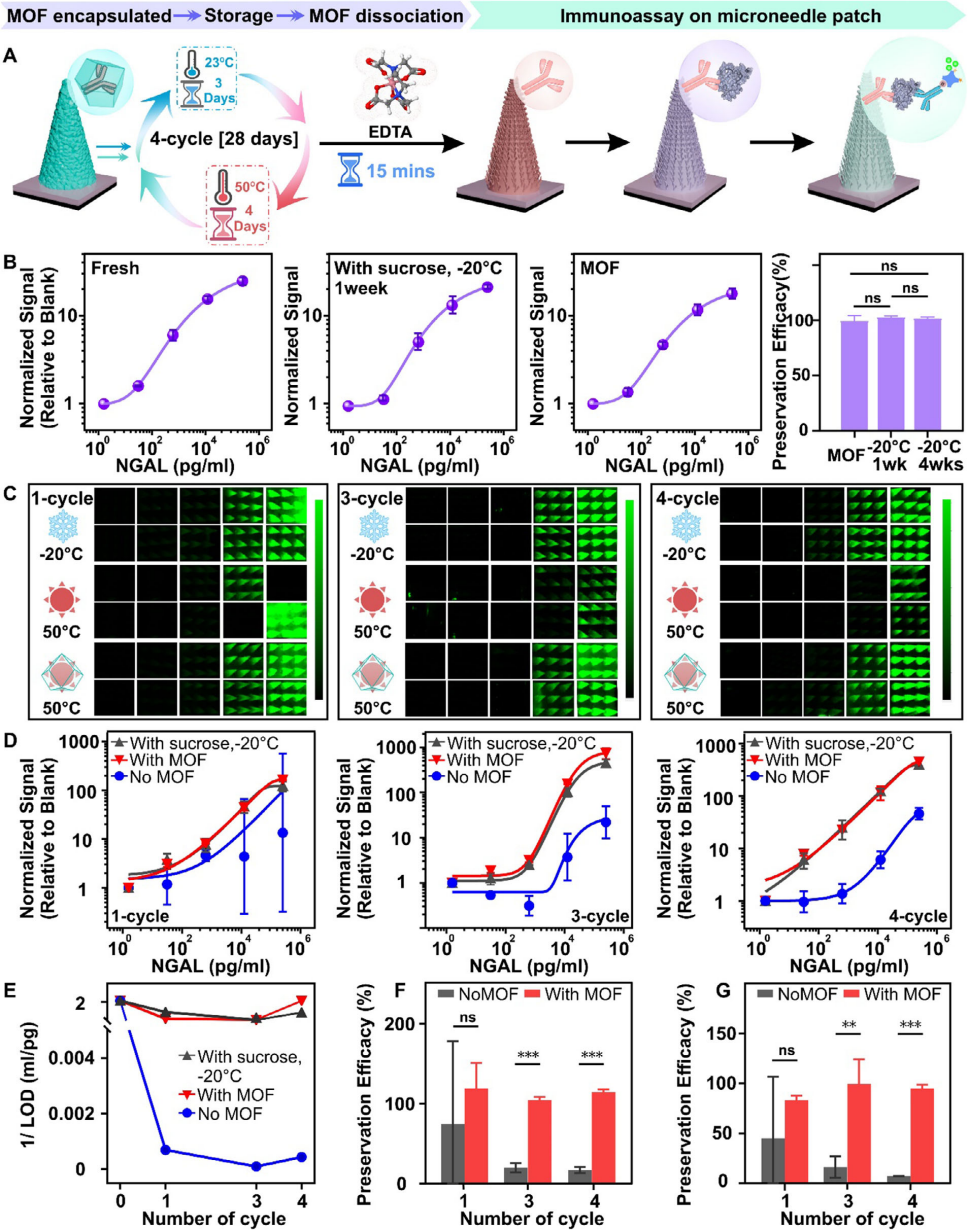
MOF encapsulation enhances the thermal stability of microneedle based‐NGAL detection. (A) Schematic illustration depicting the experimental workflow: capture antibody‐functionalized MN patches were encapsulated with ZIF‐8, subjected to repeated temperature cycling between 23°C and 50°C for 1 cycle, 3 cycles and 4 cycles, followed by on‐demand ZIF‐8 dissociation and NGAL FLISA on MN patches. 1 cycle = 3 days at 23°C + 4 days at 50°C. (B) Standard curve of microneedle‐based NGAL FLISA after storing capture antibody‐functionalized MN under different conditions. (C) Fluorescence intensity maps, (D) standard curves, (E) LODs obtained from microneedle‐based NGAL FLISA after storing capture antibody‐functionalized MN at different thermal conditions. Preservation efficacy is calculated as the percentage of fluorescence intensities retained at the highest concentration (F) and second‐highest concentrations (G) within the linear dynamic range of the FLISA standard curves. Statistical significance was determined using unpaired *t*‐tests (*ns* = nonsignificant; *p < 0.05, **p < 0.01, ***p < 0.001). Standard deviation from three replicates across 18 microneedles within the same experiment.

To simulate real‐world conditions and resource‐limited environments where refrigeration may be inaccessible, antibody‐functionalized MN patches, with and without ZIF‐8 encapsulation, were exposed to rigorous thermal cycling conditions (1 cycle = 3 days at 23°C + 4 days at 50°C) for up to 4 cycles (4 weeks in total). Assay performance was evaluated after normalizing to blank intensities, allowing comparison across different storage conditions by correcting for baseline variations. After 4 cycles of thermal stress, ZIF‐8‐protected MN patches retained excellent sensitivity, exhibiting a limit of detection (LOD) of 0.5 pg mL^−^^1^, closely matching the refrigerated positive control (LOD 1.2 pg mL^−^^1^) (Figure 5C–E). In contrast, MN patches without ZIF‐8 protection exhibited pronounced degradation in analytical performance, with the LOD increasing to 2290 pg mL^−1^, representing an approximately 2000‐fold reduction in sensitivity compared with MOF‐protected MN patches. For clarity, sensitivity was plotted as 1/LOD in the figure, where higher values indicate better assay performance (Figure 5E). In addition to preserving sensitivity, ZIF‐8 encapsulation effectively maintained the broad dynamic range essential for detecting physiologically and pathologically relevant NGAL concentrations. This capability is particularly critical for in vivo monitoring of acute kidney injury, where NGAL levels can fluctuate over several orders of magnitude depending on severity and recovery stages of renal stress [6, 9, 10]. To evaluate long‐term performance, preservation efficacy was quantified as the percentage of fluorescence intensity retained at the highest and second‐highest NGAL concentrations relative to the refrigerated control. After 4 weeks of thermal cycling between 23°C and 50°C, corresponding to a cumulative 16 days of exposure at 50°C combined with repeated temperature fluctuations, ZIF‐8‐encapsulated MN patches retained 94 ± 4% of their fluorescence intensity, whereas unprotected MN patches displayed only 7 ± 0.4% signal retention (Figure 5F,G). This cycling design serves as an accelerated thermal challenge and demonstrates the excellent protective effect of ZIF‐8 encapsulation on antibody‐functionalized MN patches. To further evaluate environmental robustness beyond temperature alone, MOF‐encapsulated MN patches were exposed to UV irradiation for 2 and 4 h, followed by EDTA‐mediated ZIF‐8 dissociation and NGAL FLISA analysis. The UV‐treated MOF@MN patches retained nearly complete fluorescence response compared with the fresh control, indicating that ZIF‐8 encapsulation preserved antibody bioactivity under these additional environmental stress conditions (Figure S16). Collectively, these results demonstrate that in situ ZIF‐8 encapsulation confers robust protection to antibody‐functionalized MN patches, enabling quantitative NGAL detection after prolonged thermal cycling and UV exposure. This excellent environmental stability positions the MOF@MN platform as a promising tool for reliable, cold‐chain‐free diagnostics and longitudinal biomarker monitoring in decentralized and resource‐limited healthcare settings.

### 2.6 Quantification of NGAL Using MOF‐Stabilized Microneedle Sensors After Ambient Transport

To evaluate the real‐world applicability of MOF@MN platform, we examined its analytical performance in quantifying NGAL in dermal ISF under operationally challenging conditions representative of decentralized healthcare environments (Scheme 1). Specifically, antibody‐functionalized MN patches were in situ encapsulated with ZIF‐8, exposed to thermal stress at 50°C for 3 days, and shipped between Missouri and Texas under standard ambient conditions for 7 days, simulating high‐temperature exposure and nonrefrigerated transport common in resource‐limited settings. Upon arrival, ZIF‐8 coatings were dissociated, and the MN were used for in vivo NGAL detection in a mouse model of LPS‐induced AKI (Figure 6A).

**FIGURE 6:**
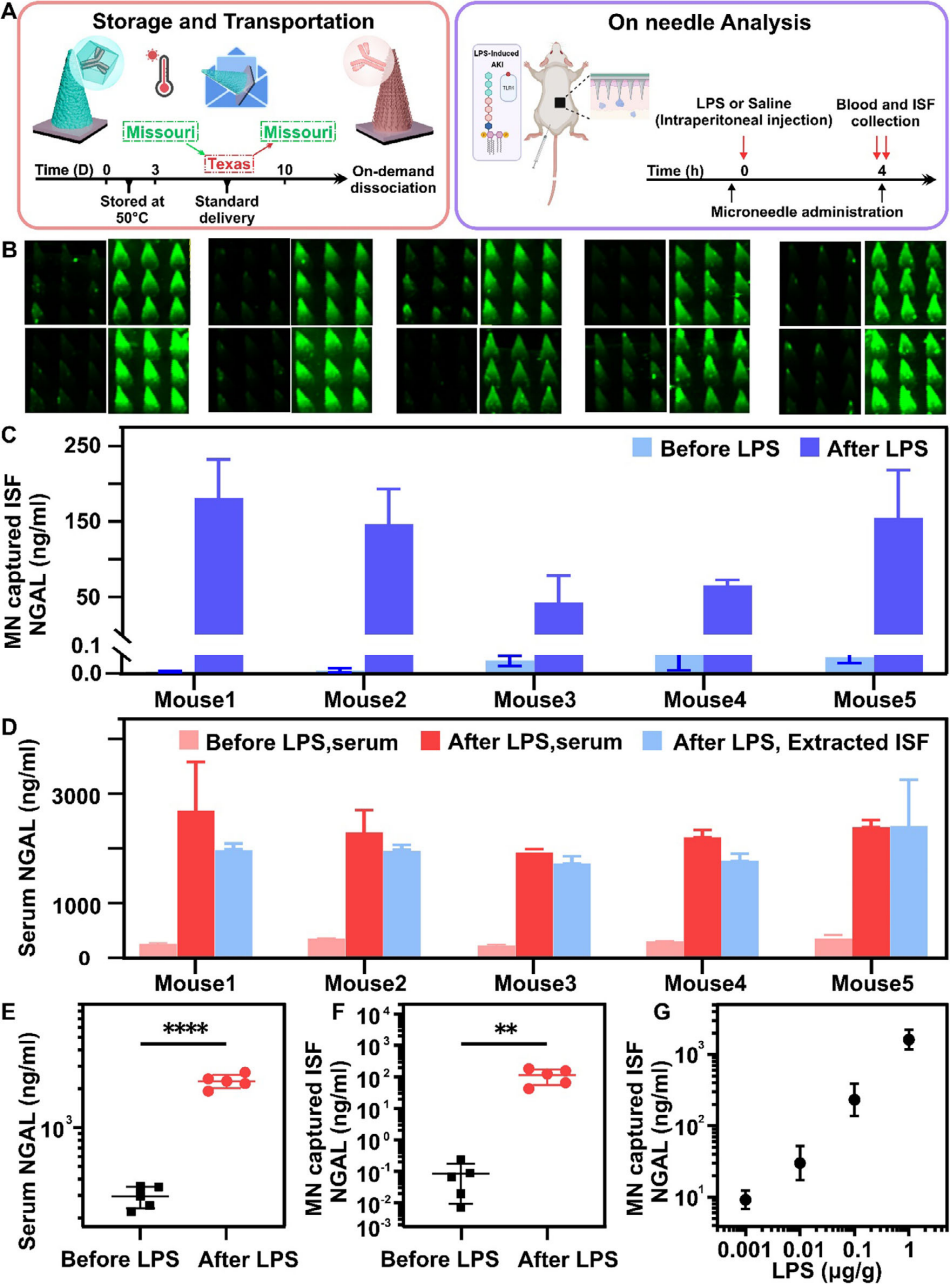
Quantification of NGAL in an LPS‐induced AKI mouse model using MOF‐encapsulated MN following real‐world ambient transportation. (A) Schematic of the validation workflow: capture antibody‐functionalized MN patches were in situ encapsulated with ZIF‐8 and subjected to elevated temperature stress (50°C for 3 days), followed by transportation between Missouri and Texas under uncontrolled ambient standard conditions (7 days in total). Post‐transportation, the ZIF‐8 coatings were removed, and in vivo NGAL detection was performed in an LPS‐induced AKI mouse model. (B) Fluorescence images corresponding to mouse NGAL captured on the microneedles before and after 4 h of LPS injection. (C) Quantification of NGAL in dermal ISF using MOF@MN patches and (D) corresponding fluorescence intensity maps before and 4 h post‐LPS injection. (E) Statistical analysis of serum NGAL levels before and after LPS injections. (F) Statistical analysis of MN‐captured ISF NGAL concentrations before and after LPS injection, confirming a significant postinjection NGAL elevation. Statistical significance was determined using unpaired *t*‐tests (ns = non‐significant; *p < 0.05, **p < 0.01, ***p < 0.001, ****p < 0.0001). (G) MN captured ISF NGAL concentrations increase with LPS dose in a dose‐dependent manner, suggesting ISF as a feasible, less invasive alternative biofluid for NGAL measurement.

To model subclinical or early‐stage AKI, BLAB/C mice received a moderate LPS dose (0.1 µg/g body weight), which induces early molecular and cellular kidney stress without significantly altering sCr and BUN levels (Figure 1). Recovered MOF@MN patches were applied to the ventral skin for 1 min both before and 4 h post‐LPS injection. Fluorescence imaging revealed a pronounced increase in signal intensity following LPS exposure (Figure 6B), and quantitative analysis confirmed a significant elevation in dermal ISF NGAL concentration from 0.08 ± 0.11 to 147.5 ± 72.8 ng mL^−1^ after exposure (Figure 6C). These results demonstrate that the MOF@MN platform can sensitively detect early renal injury even after prolonged ambient storage and transportation. Although NGAL concentrations measured via MN patches (147.5 ± 72.8 ng/mL) were lower than those detected in serum (2298.7 ± 278.8 ng/mL) or extracted ISF (1963.5 ± 271.2 ng/mL) (Figure 6C–F), the biomarker trends were consistent across sample types. The discrepancy in absolute concentration likely reflects inherent differences in capture dynamics and tissue diffusion: microneedle sampling occurs within dense dermal matrices during a brief (1‐min) administration, whereas serum assays involve dilute, homogeneous fluids with longer incubation duration. Importantly, these methodological distinctions did not affect trend fidelity, NGAL levels measured by MOF@MN devices remained predictive of disease severity and stage.

To further validate analytical reliability and dynamic responsiveness of the platform, we established a dose‐response relationship between increasing LPS doses (0.01, 0.1, and 1 µg/g) and the corresponding NGAL concentrations captured in dermal ISF by MN patches. As observed in earlier sections (Figure 1G,H) and reaffirmed here, NGAL concentrations elevated in a dose‐dependent manner, from 33.3 ± 19.1 ng mL^−1^ at low LPS dose (0.1 µg/g) to 1676.9 ± 515.1 ng mL^−1^ at the highest LPS dose (1 µg/g) (Figure 6G; Figure S16). These results confirm that the MN patches reliably recapitulate AKI progression and maintain quantitative agreement with serum and extracted ISF trends (Figure 1G,H).

Collectively, these results demonstrate that the MOF@MN patches preserve analytical performance and biosensing fidelity even after exposure to extreme thermal stress and uncontrolled ambient transport. The platform reliably captured physiologically relevant NGAL dynamics in dermal ISF, accurately reflecting early renal stress and disease progression across varying LPS doses. This experiment establishes the operational robustness and real‐world feasibility of the MOF@MN system, demonstrating that antibody‐functionalized MN patches retain quantitative precision and functional stability under environmental challenges representative of decentralized or at‐home monitoring. Beyond NGAL and AKI, this modular sensing strategy offers a broadly adaptable framework for developing portable, thermally resilient, and high‐sensitive biosensors applicable to diverse disease biomarkers and point‐of‐care settings.

### 2.7 Conclusion and Discussion

The integration of MOF encapsulation with microneedle biosensing establishes a robust and field‐deployable approach for quantitative molecular diagnostics. By leveraging in situ growth of ZIF‐8 directly on antibody‐functionalized MN, the MOF@MN platform overcomes a longstanding barrier in biosensor translation, the instability of surface‐immobilized biomolecules during storage and transport. The conformal ZIF‐8 nanocoating protects immobilized antibodies from thermal and environmental degradation while preserving biomolecular accessibility for rapid and quantitative capture of analytes from interstitial fluid. Therefore, antibody functionality and assay sensitivity are retained under prolonged temperature stress and environmental fluctuations. This design transforms microneedle‐based sensing into a cold‐chain‐independent technology, capable of reliable operation outside controlled laboratory settings. Validated across murine model and human subjects, the MOF@MN system demonstrated quantitative accuracy, biocompatibility, and translational readiness. In a lipopolysaccharide‐induced model of kidney injury, NGAL levels measured in dermal ISF closely paralleled serum and histological profiles, capturing early renal stress that precedes changes in conventional renal markers, even after high temperature exposure and ambient transportation. In human subjects, microneedle‐based ISF quantification provided reproducible, pain‐free detection and exhibited strong correlation with plasma concentrations, confirming analytical reliability and physiological relevance. Together, the results underscore the potential of the MOF@MN system for point‐of‐care diagnostics, home‐based monitoring, and deployment in resource‐limited settings.

This convergence of molecular preservation, material engineering, and minimally invasive sensing establishes a versatile diagnostic framework that bridges laboratory‐grade precision with field‐ready usability. Beyond NGAL and kidney injury, the modularity of MOF encapsulation and the adaptability of microneedle formats enable generalization to diverse classes of biomolecules, including proteins, metabolites, and nucleic acids, thereby broadening the scope of decentralized biodiagnostics. Collectively, this work lays the foundation for next‐generation biosensors that combine analytical robustness with operational independence, enabling reliable, quantitative, and patient‐centered health monitoring across clinical and decentralized environments.

Although this work establishes the foundation for cold‐chain‐independent microneedle biosensing, several aspects warrant further investigation to advance clinical translation. Protein biomarker distributions in interstitial fluid remain insufficiently characterized relative to blood, and elucidating their concentration dynamics and diffusion kinetics will be critical for defining clinically actionable ISF thresholds. The current study focuses on NGAL as a representative biomarker; however, translation to clinical practice will benefit from multiplexed panels encompassing additional kidney injury markers to enhance diagnostic specificity and rule out confounding physiological factors that influence single‐analyte measurements.

## Author Contributions

S.S. and Y.W. conceived and supervised the project. S.S., L.T., Y.W. and Y.L. designed the experiments. Y.W., A.D., and Z.W. fabricated the microneedle patches and magnetic nanoparticles. Y.W., A.D., C.L., and Y.M.C. carried out the animal studies. L.T. and Y.L. conducted the human studies. Y.W., C.L., G.K., and J.J.M. performed the bioassays. Y.W. acquired fluorescence images, SEM images, AFM images and ATR‐FTIR spectra. S.B. acquired AFM images of samples immobilized on silicon. S.S., L.T., Y.M.C., J.J.M., Y.W., and Y.L. wrote the manuscript. All authors discussed the results and commented on the manuscript.

## Conflicts of Interest

S.S. and J.J.M. are cofounders and shareholders of Brightest Bio (DBA Auragent Bioscience). S.S. and J.J.M. are inventors of the plasmonic‐fluor technology, which has been licensed by the Office of Technology Management at Washington University in St. Louis to Brightest Bio. These potential conflicts of interest have been disclosed and are being managed by Washington University in St. Louis. The other authors declare no competing interests.

## Supporting information




**Supporting File**: adma74423‐sup‐0001‐SuppMat.docx.

## Data Availability

The data that support the findings of this study are available from the corresponding author upon reasonable request.
