# Supplementary material for: Metal–Organic Framework‐Preserved Thermostable Microneedle Patch for Minimally Invasive Detection and Monitoring of Kidney Dysfunction
Source: Adv Mater. 2026 Aug 5;38(55):e74423. doi: 10.1002/adma.74423 (PMC13629254; doi:10.1002/adma.74423)
Supplement: Supplementary file 1 [file ADMA-38-e74423-s001.docx]

**Metal-Organic Framework-Preserved Thermostable Microneedle Patch for Minimally Invasive Detection and Monitoring of Kidney Dysfunction**

Yixuan Wang^1‡^, Ying Liu^2‡^, Avishek Debnath^1^, Chuang Li^3^, Zheyu Wang^1^, Gayathri Kondepati^1^, Saketh Balmoori^1^, Jeremiah Morrissey^1^, Ying Maggie Chen^3,4*^, Limei Tian^2*^, and Srikanth Singamaneni^1^*

^1^ Department of Mechanical Engineering and Materials Science, Institute of Materials Science and Engineering, Washington University in St. Louis, St. Louis, MO 63130, USA

^2^ Department of Biomedical Engineering, Texas A&M University, College Station, TX 77845, USA

^3^ Division of Nephrology, Department of Internal Medicine, Washington University School of Medicine, St. Louis, MO 63110, USA

^4^ St. Louis Veteran Affairs Medical Center, St. Louis, MO 63110, USA

*To whom correspondence should be addressed: [singamaneni@wustl.edu](mailto:singamaneni@wustl.edu) (SS), [ltian@tamu.edu](mailto:ltian@tamu.edu) (LT) and [ychen32@wustl.edu](mailto:ychen32@wustl.edu) (YC)

.‡ These authors contributed equally to this work

**Experimental Section**

*Animal studies*

All procedures have been approved (protocol ID: 24-0116)  by the Institutional Animal Care and Use Committee (IACUC) at Washington University in St. Louis. Mice were housed in an institutional animal facility at 21-23 °C and 45-50% relative humidity under a 12-hour light-dark cycle, with lights on from 07:00-19:00. Food and water were available ad libitum throughout the studies. Animal experiments were conducted and reported in accordance with institutional animal-care guidelines and the ARRIVE guidelines.

*Synthesis of magnetic nanoparticles.*

Magnetite (Fe_3_O_4_) nanoparticles were prepared through a modified polyol synthesis, following previously reported procedures with slight adjustments. Briefly, 1.05 g of iron (III) chloride hexahydrate (FeCl₃·6H₂O; Sigma-Aldrich, 236489) was dissolved in a mixture of 21 mL ethylene glycol (Sigma-Aldrich, 102466) and 7 mL ethanolamine (Sigma-Aldrich, 398136) under continuous stirring to form a homogeneous light-brown solution. After stirring for 30 min, 1.17 g of polyethylene glycol (PEG; Sigma-Aldrich, P3640) and 4.64 g of sodium acetate trihydrate (Sigma-Aldrich, S8625) were introduced into the solution while maintaining vigorous agitation. The mixture was then transferred to a Teflon-lined stainless-steel autoclave and maintained at 200 °C for overnight. Upon completion, the reaction mixture was cooled to room temperature, and the resulting Fe₃O₄ nanoparticles were collected magnetically and washed three times with ethanol and deionized water to remove residual reagents. The purified nanoparticles were subsequently redispersed in dichloromethane for storage and further use.

*Fabrication of microneedles.*

Microneedle (MN) arrays were fabricated using silicone molds containing conical cavities (Blueacre Technology). Each microneedle measured 629 µm in height with a base diameter of 300 µm and a center-to-center spacing of 600 µm. A 20% (w/v) solution of polystyrene in dichloromethane was prepared and dispensed onto the silicone mold surface. The mold was subjected to vacuum from the base to generate negative pressure, allowing the polymer solution to fill each cavity completely. The mold was then kept at ambient temperature for at least 4 hours to facilitate solvent evaporation and form solidified polystyrene MN. To prepare magnetically responsive patches, Fe₃O₄ nanoparticles were incorporated into the polystyrene matrix. Specifically, Fe₃O₄ nanoparticles were dispersed in a 10% (w/v) polystyrene–dichloromethane solution at a final nanoparticle concentration of 10 mg mL⁻¹. The resulting mixture was applied to the back side of the microneedle patch under vacuum and subsequently air-dried at room temperature for 5 hours. After drying, the microneedle patches were carefully demolded and cut into small square sections for further use. All fabrication and storage processes were performed under sterile conditions to ensure biocompatibility and prevent contamination.

*Synthesis of AuNR particles.*

AuNRs were synthesized using a seed-mediated growth method.^51-53^ To prepare the seed solution, 0.6 ml of freshly prepared, ice-cold 10 mM sodium borohydride (NaBH_4_) (Sigma Aldrich, 71321) solution was rapidly added into a solution containing 9.75 ml 100 mM cetyltrimethylammonium bromide (CTAB) (Sigma Aldrich, H5882) and 0.25 ml 10 mM gold chloride (HAuCl_4_) (Sigma Aldrich, 520918) under vigorous stirring at room temperature for 10 min. The solution color changed from yellow to brown, indicating the formation of gold seed. The growth solution was prepared by sequentially adding 2 ml of 10 mM HAuCl4, 40 ml of 100 mM CTAB, 0.65 ml of 10 mM silver nitrate (AgNO_3_) (Sigma Aldrich, 204390), 0.8 ml of 1 M hydrochloric acid (HCl) (Sigma Aldrich, H9892) and 0.32 ml of 100 mM ascorbic acid (Sigma Aldrich, A92902). The mixture was gently homogenized and 0.5µl of the seed solution was introduced into the growth solution and left undisturbed in dark at room temperature for 20 hours to allow the growth of gold nanorod. The resulting AuNRs were harvested by centrifugation at 7,000 rpm for 30 minutes to remove the supernatant. The pellet (AuNRs) was re-dispersed in nanopure water for further use.

*Immobilize IgG-AuNR on silicon substrate.*

Conjugation of AuNRs with rabbit immunoglobulin G (IgG) was performed through a two-step coupling process.^54^ Briefly, IgG molecules were first modified with a bifunctional polyethylene glycol linker (COOH-PEG-SH, 5 kDa) via carbodiimide-mediated amide coupling between the carboxyl groups of PEG and the amine groups on IgG. The resulting thiol-terminated IgG-PEG-SH conjugates were subsequently immobilized onto the AuNR surface through covalent Au–S bonding. (3-mercaptopropyl)triethoxysilane (MPTS)-modified silicon substrates were incubated with the AuNR–IgG conjugate suspension for 2 hours at room temperature to enable thiol-mediated anchoring of the nanostructures. The substrates were then thoroughly rinsed with ultrapure water to remove non-specifically adsorbed conjugates and gently dried under nitrogen flow before further use.

*Mouse NGAL FLISA on microneedle and on microtiter plate.*

Fluorescence-linked immunosorbent assay (FLISA) for mouse neutrophil gelatinase-associated lipocalin (NGAL) was performed using the Mouse NGAL DuoSet ELISA kit (R&D Systems, DY1857) with modifications for microneedle (MN) substrates. MN patches were placed in a 24-well plate (Corning, 3738) mounted on a magnetic plate separator (Luminex, CN-0269-01) to ensure firm contact with the well bottom throughout incubation and washing steps.

Each MN patch was first immersed in 0.2% poly(ethyleneimine) (PEI, Sigma-Aldrich, P3143) solution and washed three times with nanopure water. The PEI-coated MN was then immersed with 1 mL of capture antibody solution and incubated overnight at room temperature. After incubation, patches were washed three times with PBS containing 0.05% Tween-20 (PBST) and blocked with 1.5 mL reagent diluent (PBS supplemented with 3% bovine serum albumin, 0.2 μm filtered) for 2 hours at room temperature. Following blocking, 1 mL of serially diluted NGAL standards or sample solutions was added and incubated for 20 minutes at room temperature. Patches were washed three times with PBST and incubated with biotinylated detection antibody for 2 hours. After washing, patches were incubated with IRDye 800CW-labeled streptavidin (LI-COR, 926-32230) for 20 minutes, followed by three washes each with PBST and nanopure water. Patches were imaged using a LI-COR CLx fluorescence imager.

Parallel NGAL FLISA assays were performed on 96-well plates using 100 μL capture antibody solution (2 μg mL⁻¹ in PBS) and first incubated overnight at room temperature. After incubation, microtiter plates were washed three times with PBST and blocked with 200 μL reagent diluent (PBS supplemented with 3% bovine serum albumin, 0.2 μm filtered) for 1 hour at room temperature. Following blocking, serially diluted NGAL standards or sample solutions were added and incubated for 2 hours at room temperature. Plates were washed three times with PBST and incubated with biotinylated detection antibody for 2 hours. After washing, patches were incubated with IRDye 800CW–labeled streptavidin (LI-COR, 926-32230; 5 μg mL⁻¹) for 20 min, followed by three washes each with PBST. Plates were imaged using a LI-COR CLx fluorescence imager. The concentration of the protein in unknown samples was determined using standard curve fit with a 4-parameter logistic model.

*In Situ Sampling and Quantification of NGAL in Human ISF.*

MN patches were functionalized with human NGAL capture antibodies, thoroughly rinsed with PBST, and blocked with 3% BSA in PBS for 1 hour at room temperature. Antibody-functionalized patches underwent bacterial endotoxin testing following the complete functionalization process. MN patches were incubated overnight in endotoxin-free water at room temperature, and the eluate was analyzed using the Kinetic Chromogenic Limulus Amebocyte Lysate (LAL) assay (Thermo Scientific, 88282). The measured endotoxin level was expressed in endotoxin units (EU) per device. Before application, the ventral forearm skin of human volunteers was cleaned with 70% isopropyl alcohol and allowed to dry. Each MN patch was applied to the skin for 5 min using a spring-loaded applicator to ensure uniform insertion and consistent ISF sampling. After removal, patches were washed with PBST and sequentially incubated with biotinylated detection antibodies and plasmonic fluor-800CW nanolabels^39^, followed by three additional PBST washes. The resulting fluorescence signal was acquired using a LI-COR Odyssey M Imaging System (800 nm channel; focus offset 1.3 mm; resolution 21 μm).

For comparison, fingertip blood samples were collected using a sterile 21G lancet, and plasma was separated by centrifugation. Plasma NGAL concentrations were quantified by plasmon-enhanced FLISA (p-FLISA)^39^ using identical reagent concentrations and incubation parameters as the on-needle assay. All studies involving human participants were approved by the Texas A&M University Institutional Review Board (protocol ID: IRB 2019-0811F).

*MOF encapsulation on MN surface and IgG-AuNR surface.*

MN patches functionalized with NGAL capture antibodies were rinsed three times with nanopure water to remove residual salts and unbound biomolecules, then immersed in 1 mL of 2 wt% polyvinylpyrrolidone (PVP) solution for 30 minutes at room temperature to promote MOF growth. After removing excess PVP, each MN patch was transferred into a freshly prepared ZIF-8 precursor solution composed of 2-methylimidazole (160 mM) and zinc acetate (40 mM) aqueous solutions mixed rapidly at a molar ratio of 4:1. The mixed precursor was immediately added to the wells containing MN patches to initiate *in situ* crystallization of ZIF-8 on the antibody-coated surfaces. The reaction was maintained for 4 hours at room temperature (23 °C) to allow uniform deposition of the conformal ZIF-8 layer. Following incubation, the precursor solution was removed, and the MOF-encapsulated MN were rinsed thoroughly with nanopure water and stored at ambient conditions or subjected to thermal stress as described in the main text.

In parallel, an identical ZIF-8 growth protocol (precursor concentrations, reaction time, and temperature) was performed on IgG-Au nanorod (AuNR)coated silicon wafers to evaluate the morphological consistency across different substrates.

*Antibody Recovery and FLISA on Microneedle Patches.*

To recover antibodies encapsulated within ZIF-8 coatings, MN patches were immersed in 1.5 mL of MOF dissociation buffer consisting of 0.1 M phosphate buffer supplemented with 100 mM EDTA and 0.05% Tween-20 (pH 5.1). The samples were gently agitated on an orbital shaker for 30 minutes at room temperature to facilitate complete chelation of Zn²⁺ ions and dissolution of the ZIF-8 layer. After dissociation, the buffer was aspirated, and the MN patches were washed three times with PBST to remove residual chelating agents and dissolved components. The recovered, antibody-functionalized MN patches were subsequently blocked with 1.5 mL of reagent diluent (PBS containing 3% bovine serum albumin, 0.2 µm filtered) for 2 hours at room temperature to minimize non-specific binding. Following blocking, the patches were subjected to the fluorophore-linked immunosorbent assay (FLISA) using identical reagents and incubation conditions as described in the MN FLISA section.

*Bacterial Endotoxin Quantification of Microneedle Patches.*

The endotoxin level associated with MN patches was evaluated to ensure biosafety, particularly following MOF encapsulation and dissociation. MOF first *in situ* growth on NGAL capture antibody-immobilized MN surface and then use MOF dissociation buffer to remove MOF layer. After MOF encapsulation and dissociation, MN patches were immersed in endotoxin-free water and incubated overnight at room temperature to allow potential surface-associated endotoxins to diffuse into the solution. After incubation, the eluate was collected and analyzed for bacterial endotoxin content using the Kinetic Chromogenic Limulus Amebocyte Lysate (LAL) assay (Thermo Scientific, 88282) according to the manufacturer’s protocol. The detected endotoxin concentration was expressed in endotoxin units (EU) per patch, providing a quantitative assessment of residual pyrogenic contamination on the MN surface.

*Assessment of Systemic Toxicity of MN Patches.*

To evaluate the systemic biocompatibility of the MN patches, healthy mice were administered with MN patches after MOF encapsulation and dissociation. Following MN application, major organs including the heart, lungs, liver, kidneys, and spleen were collected for histopathological evaluation. All tissues were fixed in 4% neutral buffered formalin, processed through standard dehydration and paraffin embedding procedures, and sectioned for hematoxylin and eosin (H&E) staining. Histological processing and staining were conducted by the Musculoskeletal Histology and Morphometry Core Facility at Washington University in St. Louis. The stained tissue sections were examined under bright-field illumination using a Zeiss AxioScan 7 microscope to assess tissue morphology, inflammatory infiltration, necrosis, or other pathological alterations indicative of systemic toxicity.

*Material Characterization.*

SEM images were obtained using Thermo Scientific Quattro S Environmental Scanning Electron Microscope (ESEM). Atomic force microscopy (AFM) images were collected by Dimension 3000 AFM (Digital instruments) in light tapping mode. Attenuated total reflection Fourier transform infrared spectra (ATR-FTIR) were obtained using a Nicolet iS10 spectrometer in the range of 400 - 4000 cm^-1^ at a resolution of 0.48 cm^-1^, averaging 100 scans. The Raman spectra was obtained using a Renishaw inVia confocal Raman spectrometer mounted on a Leica microscope with a 20X objective and a 785 nm wavelength diode laser was employed as the illumination source. Raman

*Statistics.*

Statistical analyses were performed using GraphPad Prism 10. Differences between two independent groups were assessed using an unpaired two-tailed t-test. Statistical significance was determined at a 95% confidence level (P < 0.05). Data are reported as mean ± s.d. For bioassay calibration curves, four-parameter logistic were used to model the standard curves and estimate the limit of detection (LOD). The LOD was defined as the analyte concentration corresponding to the mean fluorescence intensity of the blank plus three times its standard deviation (mean + 3σ).

**Figures**

Figure S1. Temporal dynamics of serum creatinine (sCr) at 0.1 and 1 µg/g LPS dosages compared with saline controls.

Figure S2. SEM images of microneedle patches from (A) top view, (B) and (C) front view.

Figure S3. Confocal fluorescence microscopy image confirming uniform adsorption of antibodies on polystyrene microneedles.

Figure S4. (A) Fluorescence intensity maps corresponding to pristine microneedles, BSA coated microneedles and biotinylated NGAL capture antibody coated microneedles exposed to IRDye 800CW-labeled streptavidin. (B) Plot showing that BSA coated microneedles exhibit extremely weak fluorescence intensity, indicating the successful coating of the NGAL capture antibody on the polystyrene microneedle surface. n=3

Figure S5. Fluorescence intensity maps of MN patches following exposure to known concentrations of NGAL spiked in diluted mouse ISF matrix.

Figure S6. H&E staining of mouse dermis and epidermis layer (A) without and (B) with microneedle administration.

Figure S7. SEM images of MN surface morphology and blood-cell contamination after skin administration. SEM images of (A) the standard 0.6 mm MN patch used in this study before and after mouse skin administration and (B) the 1.5 mm over-penetrating MN patch.

.

Figure S8. (A) Fluorescence intensity maps of microneedle patches and (B) a plot of fluorescence intensity with and without administration on mouse skin, showing a 19% loss of antibodies due to shear forces during skin penetration.

.

Figure S9. Standard curve of LAL Chromogenic Endotoxin Quantitation Kit.

Figure S10. AFM scratch measurements of MOF grown on polystyrene-coated silicon substrates. (A) 2 wt% polystyrene film spin-coated on a silicon wafer. (B) NGAL capture antibody layer adsorbed on the PS surface. (C) Antibody-functionalized PS surface encapsulated by 30 nm-MOF. (D) Antibody-functionalized PS surface encapsulated by 300 nm-MOF.

Figure S11. Raman spectra confirming the formation and dissociation of ZIF-8 and its interaction with antibodies-functionalized surface.

Figure S12. Complete MOF coverage on microneedle surfaces. A stitched montage of 56 SEM images demonstrates continuous, MOF coating across the entire microneedle array without observable gaps or defects.

Figure S13. Morphology-dependent MOF encapsulation on microneedle tips. Representative SEM images of (A) Pristine microneedle (MN) tip, (B) MN tip conformally encapsulated by a ~30 nm MOF (30nm-MOF), (C) MN tip coated with ~300 nm rhombic dodecahedral ZIF-8 crystals (300 nm-MOF) and (D) Cross-sectional SEM image showing 300 nm rhombic dodecahedral ZIF-8 crystals grown on the antibody-functionalized MN surface.

Figure S14. (A) Fluorescence images of microneedle patches and (B) preservation efficacy without and with MOF encapsulation under different conditions. Statistical significance was determined using unpaired t-tests (ns = non-significant; ***p<0.001).

Figure S15. Standard curve of LAL Chromogenic Endotoxin Quantitation Kit. Error bars represent standard deviation. N=2 repeated tests.

|  | **OD 405** | **Concentration (EU/ml)** |
| --- | --- | --- |
| **Sample 1** | **0.1239** | **0.054** |
| **Sample 2** | **0.1221** | **0.052** |
| **Average** |  | **0.053** |

Table S1. Individual data point of endotoxin test for microneedles after MOF encapsulation and dissociation process.

Figure S16. (A) Fluorescence images of microneedle patches and (B) Fluorescence intensity of MN at different conditions. Statistical significance was determined using unpaired t-tests (ns = non-significant).

Figure S17. Fluorescence intensity maps and quantitative comparison of serum and microneedle-based NGAL detection under LPS administration.
